# Prostate MRI: a national survey of Urologist’s attitudes and perceptions

**DOI:** 10.1590/S1677-5538.IBJU.2015.0235

**Published:** 2016

**Authors:** Brandon J. Manley, John A. Brockman, Valary T. Raup, Kathryn J. Fowler, Gerald L. Andriole

**Affiliations:** 1 Division of Urologic Surgery, Department of Surgery, Washington University School of Medicine St. Louis, USA;; 2 Mallinckrodt Institute of Radiology, Washington University School of Medicine, St. Louis, MO, USA

**Keywords:** Prostate, Magnetic Resonance Imaging, Prostatic Neoplasms

## Abstract

**Introduction:**

The use of multi-parametric (MP) MRI to diagnose prostate cancer has been the subject of intense research, with many studies showing positive results. The purpose of our study is to better understand the accessibility, role, and perceived accuracy of MP-MRI in practice by surveying practicing urologists.

**Materials and Methods:**

Surveys were sent to 7,400 practicing American Urological Association member physicians with a current email address. The survey asked demographic information and addressed access, accuracy, cost, and role of prostate MRI in clinical practice.

**Results:**

Our survey elicited 276 responses. Respondents felt that limited access and prohibitive cost of MP-MRI limits its use, 72% and 59% respectively. Academic urologists ordered more MP-MRI studies per year than those in private practice (43.3% vs. 21.1%; p<0.001). Urologists who performed more than 30 prostatectomies a year were more likely to feel that an MP-MRI would change their surgical approach (37.5% vs. 19.6%, p-value=0.002). Only 25% of respondents agreed or strongly agreed that MP-MRI should be used in active surveillance. For patients with negative biopsies and elevated PSA, 39% reported MP-MRI to be very useful.

**Conclusions:**

Our study found that MP-MRI use is most prominent among practitioners who are oncology fellowship-trained, practice at academic centers, and perform more than 30 prostatectomies per year. Limited access and prohibitive cost of MP-MRI may limit its utility in practice. Additionally, study participants perceive a lack of accuracy of MP-MRI, which is contrary to the recent literature.

## INTRODUCTION

Prostate cancer is the second leading cause of cancer-related death in men, claiming almost 30.000 lives in 2013 (1). It is a heterogeneous disease with differences in biological aggressiveness, and the natural history of prostate cancer is highly variable from patient to patient. It has been shown that over half of those diagnosed with prostate cancer will die of other causes (2). In light of the United Service Preventative Task Force recommendation against the use of prostate specific antigen (PSA) for cancer screening, as well as several studies demonstrating no survival advantage with aggressive treatment, there has been a growing interest in the use of active surveillance in prostate cancer management (3). As the treatment algorithm for prostate cancer continues to evolve, there has been increased emphasis on the use of multi-parametric magnetic resonance imaging (MP-MRI) to aid in the diagnosis and management of prostate cancer, especially within the active surveillance population.

MRI provides high-resolution anatomic detail via T2 weighted images and allows for functional assessment of the prostate. Dynamic contrast enhanced imaging measures the vascularity of tumors, and the vascular nature of prostate cancer shows both increased uptake and washout of gadolinium contrast when given intravenously (4). Diffusion weighted imagining measures the diffusion of water through tissue, causing prostate cancer to exhibits reduced diffusion compared to normal prostate tissue due to its densely packed cells.

Despite demonstration of the benefit within the literature, MP-MRI has been slow to gain widespread acceptance (5). The practice patterns, experience, and attitudes of American urologists regarding the use of MRI in the management of prostate cancer have not been previously examined. The goal of our study is to characterize the opinions amongst current American urologists concerning the role of MP-MRI in prostate cancer management.

## MATERIALS AND METHODS

This study was reviewed and approved by the Institution Review Board at Washington University. A web-based survey consisting of twenty questions was developed and sent electronically to 7,400 current American Urological Association (AUA) members. All survey participants were practicing urologic physicians in the United States had current email addresses as of September 01, 2013. An initial email with a brief explanation of the study and an invitation to complete the survey was sent on September 12, 2013, and a reminder email for those who had yet to complete the survey was sent on September 25, 2013. Respondents were not required to answer all questions for submission of their survey. Each email was embedded with a personalized link to ensure that each respondent could only submit a single survey, and all responses were confidential.

Each survey was de-identified and responses were collected using our institutional RedCap electronic data-capturing tool (6). Survey questions addressing practitioner demographics included: how many years since finishing residency, post-residency training, proximity to nearest tertiary care center, structure of medical practice, practice setting, and number of prostatectomies performed yearly. Questions addressing practitioner opinion of MP-MRI included: evidence in literature supporting MP-MRI, reliability of the results of MP-MRI, and accuracy of MP-MRI measured by correlation between MP-MRI results and positive biopsies/final pathology after prostatectomy.

Categorical responses to survey questions were assessed using Chi-square test of independence analysis and Fisher’s exact test. Continuous variables were compared with the Student’s t-test, and statistical analyses were two-sided using a significance of 0.05.

## RESULTS

Our survey elicited 276 (3.8%) responses from practicing AUA member physicians. Unfortunately, not all participants answered every survey question. The demographics that were obtained from the survey question can be found in Table-1. Overall, a majority of survey participants reported ordering 1-10 MP-MRIs a year to evaluate prostate cancer. Forty-two percent of respondents agreed that there was adequate evidence within the literature supporting the use of MP-MRI in localized prostate cancer (114/272), 31% disagreed (84/272), and 27% could not decide (74/272). A summary of survey responses can be found in Table-2.

**Table 1:** Survey Demographics.

How many years since you finished residency?	Total = 276
0-5 years	54 (19.5%)
6-10 years	39 (14.1%)
11-20 years	77 (27.8%)
21-30 years	77 (27.8%)
Over 31 years	29 (10.5%)

What training, if any, did you complete after residency?	**Total = 274**

None	159 (58%)
Minimal Invasive/Endourology Fellowship	21 (7.7%)
Oncology Fellowship	66 (24.1%)
Reconstructive Fellowship	9 (3.3%)
Other	19 (6.9%)

Approximately how close is the nearest tertiary care center to your practice?	**Total = 276**

I primarily practice at a tertiary care center	134 (48.6%)
Less than 1 hour	95 (34.4%)
Less than 2 hours	32 (11.6%)
Less than 3 hours	5 (1.8%)
More than 3 hours	10 (3.6%)

What type of practice do you work in primarily?	**Total = 276**

Private Group or Solo	164 (59.2%)
Academic	91 (32.9%)
Government (VA, Military service, National Health Service)	7 (2.5%)
Other	15 (5.4%)

What type of setting do you practice in?	**Total = 276**

Urban	151 (54.7%)
Suburban	103 (37.2%)
Rural	22 (8%)

On average, how many prostatectomies do you preform yearly for Prostate cancer?	**Total = 275**

Under 10	105 (38.2%)
30-Oct	85 (30.9%)
30-100	71 (25.8%)
Over 100	14 (5.1%)

**Table 2:** Survey Responses.

There is adequate evidence supporting the use of MP-MRI to manage localized prostate cancer.	Total = 272
Agree	114 (42%)
Disagree	84 (31%)
Can not decide	74 (27%)

Access to MP-MRI limits my ability to use it in my practice.	Total = 268

Agree/Strongly agree	192 (72%)
Disagree/Strongly disagree	76 (28%)

The high cost of MP-MRI is prohibitive for its use.	Total = 263

Agree/Strongly agree	156 (59%)
Disagree/Strongly disagree	107 (41%)

MR-MPI guided biopsies are utilized in my practice.	Total = 270

Agree	91 (34%)
Disagree	179 (66%)

MP-MRI is helpful in patients with elevated PSA/abnormal prostate exam prior to biopsy.	Total = 270

Agree/Strongly agree	102 (38%)
Disagree/Strongly disagree	168 (62%)

MP-MRI is helpful in patients with negative biopsy and abnormal PSA/prostate exam.	Total = 225

Agree/Strongly agree	88 (39%)
Disagree/Strongly disagree	137 (61%)

MP-MRI is useful prior to definitive treatment with prostatectomy or radiation.	Total = 225

Agree/Strongly agree	32 (14%)
Disagree/Strongly disagree	193 (86%)

MP-MRI changes my treatment approach of intermediate/high risk prostate cancer.	Total = 253

Sometimes/often	66 (26%)
Rarely/never	187 (74%)

MP-MRI should be used in all patients for active surveillance.	Total = 276

Agree/Strongly agree	69 (25%)
Disagree/Strongly disagree	207 (75%)

How often do MP-MRI guided biopsies turn out to be positive?	Total = 233

Often/Very often	65 (28%)
Sometimes	77 (33%)
Rarely/Never	91 (39%)

How closely do MP-MRI results correlate with final pathology after prostatectomy?	Total = 233

Strong correlation	26 (11%)
Moderate correlation	145 (62%)
Weak correlation	46 (20%)
No correlation	16 (7%)

### Access

When respondents were stratified by their type of practice, there was a statistically significant increase in the reported number of MP-MRIs ordered by physicians who practice in an academic setting compared those who do not (>11 MP-MRIs/year, 43% versus 21% p=0.0001). Eighty-nine percent of respondents endorsed having local access (less than 1hr) to facilities with MP-MRI capabilities (245/276). However, when all respondents were asked if they felt that access to qualified imaging centers and radiologists limited their use of MP-MRI, 72% of respondents agreed or strongly agreed (192/268). When asked if the cost of MP-MRI was prohibitive for its use, 59% of all respondents agreed or strongly agreed (156/263).

### Role of MRI

Overall, 34% of respondents reported using MP-MRI targeted biopsies, either ultrasound fusion or cognitive techniques (91/270). When these responses were stratified by fellowship training, we found that respondents with oncology fellowship training performed significantly more MP-MRI targeted biopsies compared to those who did not (44.6% versus 30.1%, p=0.032). In our survey, 38% of all respondents found MP-MRI to be helpful in a patient with an elevated PSA or abnormal prostate exam prior to biopsy (102/270). When these responses where stratified by fellowship training, we found 48.2% of respondents with oncology training found MP-MRI somewhat or very helpful in this situation compared to 33.5% of respondents without oncology training. This difference was found to be trending towards significance with a p value of 0.057.

In patients with a negative biopsy and an elevated PSA/abnormal prostate exam, 39% of all respondents reported MP-MRI to be very helpful (88/225). Fourteen percent of respondents reported MP-MRI to be very useful when utilized prior to definitive treatment with either prostatectomy or radiation (32/225). In patients with intermediate or high risk prostate cancer who appear to be candidates for a nerve sparing prostatectomy, 26% of respondents report that getting an MP-MRI will sometimes or often change their surgical approach to a non-nerve sparing prostatectomy (66/253). When stratified based on average number of prostatectomies performed per year (<30 versus >30); we found that surgeons who perform more than 30 prostatectomies per year are more likely to change their surgical approach based on MP-MRI than those who perform less than 30/year (37% versus 20%, p=0.002).

Regarding the use of MP-MRI in active surveillance, 25% of all respondents agreed that it should be used in all patients (69/276). When asked if MP-MRI should be used to evaluate patients for active surveillance, 30.7% of respondents who were 10 years or less out of residency reported MP-MRI to be very helpful compared to 24.8% of respondents 11 years or more out of residency (30/98 versus 45/183).

### Accuracy

When asked approximately what proportion of patients with positive MP-MRI findings who subsequently undergo MP-MRI targeted prostate biopsies are found to have biopsies positive for prostate cancer, 28% reported positive biopsies “often” or “very often” (65/233), 33% reported positive biopsies “sometimes” (77/233), and 39% reported positive biopsies “rarely” or “never” (91/233). Similarly, when asked about how closely respondents find MP-MRI results to correlate with final pathology after prostatectomy, 11% reported strong correlation (26/233), 62% reported moderate correlation (145/233), 20% reported weak correlation (46/233), and 7% reported no correlation (16/233).

## DISCUSSION

The use of multi-parametric (MP-MRI) to diagnose prostate cancer has been the subject of intense research, with many studies showing positive results. To our knowledge, no other studies have attempted to better understand the accessibility, role, and perceived accuracy of MP-MRI in practice by surveying practicing urologists. We found practicing in an academic center to be strongly associated with increased use of MP-MRI (p=0.0001), which is likely due to a combination of increased access, familiarly with recent literature, and interdisciplinary efforts within academic institutions.

### Access

There is very little within the literature evaluating practitioner access to MP-MRI, particularly outside academic centers. While 89% of our respondents reported to be within one hour of a facility with MP-MRI capabilities, 72% still felt that their use of MP-MRI was limited by lack of access. While the exact reasons for this discrepancy could not be gleaned from the survey data, causes may relate to perceived lack of quality in the MP-MRI provided, difficulty with patient scheduling, or lack of strong multi-disciplinary relationship with radiologists.

Another possible reason that MP-MRI has been slow to gain wide spread popularity is that many (59% within our study) feel that the cost of these studies is prohibitive, and that issues surrounding insurance reimbursement negate the perceived value of the study. As the presence of MP-MRI becomes stronger in national and international management guidelines, insurance approval and payment for these studies may become more streamlined (7-9). Likewise, as the guidelines for active surveillance continue to evolve, MP-MRI may be incorporated into these algorithms, which will support this study as a reimbursable indication.

### Role

The use of MP-MRI in the management of prostate cancer has gained a lot of attention in recent literature; however, the appropriate use in clinical practice has not yet been established. Despite the growing body of evidence supporting the use of MP-MRI guided biopsies in lieu of saturation biopsies, many of our respondents did not echo this sediment. We found that only 42% of respondents felt that the current literature provided evidence for some role for MP-MRI in localized prostate cancer patients, while 31% disagreed and 27% could not decide. These results further prove the level of controversy surrounding MP-MRI.

MP-MRI can be used to provide guidance for tissue sampling either directly, via a cognitive approach, or through fusion software. MP-MRI guided biopsies have been shown to upgrade Gleason grade and detect otherwise undiagnosed anterior gland tumors in a significant number of patients (10-13). Despite this, only 34% of respondents reported using MP-MRI guided biopsies in their clinical practice. However, level of training was found to be associated with an increased use of MP-MRI guided biopsies. Completion of an oncology fellowship resulted in statistically significant increases in usage (p=0.032), and completion of any urologic fellowship also trended towards a significant increase (p=0.057). While this may be in part due to the fact that many of these urologists practice at academic centers, these results suggest that further training and education on MP-MRI guided biopsies may be useful to guide practice.

In intermediate or high-risk prostate cancer patients who appear to be candidates for a nerve sparing prostatectomy, MP-MRI can be helpful for surgical planning (14-15). Of our respondents, 38% found MP-MRI to be helpful when used in this capacity. We also found those urologists who reported doing more than 30 prostatectomies a year used MP-MRI for surgical or treatment planning at a higher frequency compared to their colleagues (p=0.002). It is possible that urologists with more operative experience may have increased expertise and familiarity with MP-MRI. It is also possible that they handle more complex cases, requiring the use of additional imaging modalities.

It has also been proposed to incorporate MP-MRI into active surveillance protocols (16-17). While the majority of current evidence suggests a benefit to its incorporation, there have been some studies that failed to demonstrate an improvement in the stratification of patients (18-19). In our study, only 25% of all respondents agreed or strongly agreed that MP-MRI should be used in all patients on active surveillance. In patients with a previous negative biopsy and a rising PSA or abnormal DRE there are several studies that have found MP-MRI to be helpful and this has been incorporated in to many current guidelines (7-9, 20-21). Despite this, only 39% of respondents in our study agreed that MP-MRI was helpful or very helpful for these patients. Reasons for this disconnect may be related to the dissemination of this information to practicing urologists or perceived poor performance of MRI in practice.

### Accuracy

While several studies have shown the high accuracy of MP-MRI, respondents seemed to largely express skepticism (13, 22-23). There were a large proportion of respondents who felt that MP-MRI was relatively inaccurate, with moderate-poor correlation with pathology and little positive impact on patient care. While the reason for these opinions was not elucidated in our study, this negative impression of MP-MRI may reflect differences in local radiologist and pathologist ability to interpret and correlate MP-MRI findings. It is well accepted that achieving accurate radiology-pathology correlation with MP-MRI findings is challenging, and it has been suggested that standardization of protocols is the best way to overcome these challenges (24-25). The European Society for Urogenital Radiology has developed guidelines to standardize reporting and acquisition of prostate MRIs, named the Prostate Imaging Reporting and Diagnostic System (PI-RADS) (8). Unfortunately, this system has not been universally adopted in the United States. Further standardization may help to ensure that the use of MP-MRI in the community mirrors the same accuracy and provides the same patient benefits as those reported in the literature. As urologists are able to master this learning curve, one would expect accuracy to improve.

There are several limitations to our study. The response rate of only 3.8% is clearly less than ideal, and it would have been preferable if all respondents had answered every survey question. Never the less, with 276 respondents exhibiting a wide range of demographics, we feel that the responses provide an adequate sample size. The survey was designed to provide data that could be easily analyzed, yet multiple choice answers carry the risk of being leading. It might be anticipated that the survey would have promoted a bias toward MP-MRI, yet the results indicate otherwise. Further directions for research include a survey administered to a large number of practitioners across different vendor platforms and in various institutional settings. Also, it would be helpful to obtain insight into why practitioners chose their various answers, would could be accomplished with a more in-depth amended to include the option for free response.

## CONCLUSION

Prostate MRI has been shown within the literature to be a highly useful diagnostic tool. However, our study indicates a relatively low level of support for the use of MP-MRI in clinical practice. This indicates the need for improved education, access, and standardization of treatment recommendations to address the challenges of implementing this new technology into practice. Further research is needed to confirm the favorable results of MP-MRI across different vendor platforms, in various institutional settings, and using available radiologic-pathologic correlation.
